# Effects of Lanthanum Element and Heat Treatment on Phase Formation and Magnetic Properties of SmFe_10_V_2_ Melt-Spun Ribbons

**DOI:** 10.3390/ma18102322

**Published:** 2025-05-16

**Authors:** Weiyi Ying, Zhengli Yang, Xiang Liu, Qingrong Yao, Jiang Wang

**Affiliations:** 1 Guangxi Key Laboratory of Information Materials, School of Materials Science and Engineering, Guilin University of Electronic Technology, Guilin 541004, China; yingweiyi333@163.com (W.Y.); 13140580860@163.com (X.L.); qingry96@guet.edu.cn (Q.Y.); 2 Engineering Research Center of Electronic Information Materials and Devices, Ministry of Education, Guilin University of Electronic Technology, Guilin 541004, China

**Keywords:** SmFe_10_V_2_ melt-spun ribbons, phase formation, magnetic properties

## Abstract

The effects of the La element and heat treatment on the phase composition and magnetic properties of SmFe_10_V_2_ melt-spun ribbons are studied by X-ray diffraction (XRD) and vibrating sample magnetometry (VSM). The XRD results show that the Sm_1−*x*_La*_x_*Fe_10_V_2_ (*x* = 0, 0.2, 0.4) and SmLa*_y_*Fe_10_V_2_ (*y* = 0.1, 0.2, 0.3) melt-spun ribbons are composed of the 1:12 main phase with a ThMn_12_ structure, α-Fe phase and α-La phase, and the phase formation of the SmLa_0.2_Fe_10_V_2_ melt-spun ribbon is not changed after annealing at different temperatures (700–900 °C) and times (10–60 min). The XRD results indicate that La substitution in Sm_1−*x*_La*_x_*Fe_10_V_2_ ribbons has a negative effect on the formation of the 1:12 main phase, while the addition of a small amount of La into SmLa*_y_*Fe_10_V_2_ ribbons does not affect the formation of the 1:12 main phase. The VSM measurements show that La substitution would result in decreases in the magnetic properties of Sm_1−*x*_La*_x_*Fe_10_V_2_ ribbons. With the increase in La substitution, the coercivity of the ribbon decreases to 2.7 kOe with *x* = 0.2 and 0.43 kOe with *x* = 0.4. However, the magnetic properties of SmLa*_y_*Fe_10_V_2_ ribbons with a small amount of La (*y* = 0.2) are improved. Furthermore, the experimental results for the SmLa_0.2_Fe_10_V_2_ ribbon after different heat treatments show that the magnetic properties become better initially and then become worse. Finally, the best magnetic properties (H_cj_ = 5.12 kOe, B_r_ = 6.89 kGs, M_r_/M_s_ = 0.75, (BH)_max_ = 6.78 MGOe) for the SmLa_0.2_Fe_10_V_2_ ribbon are obtained after annealing at 800 °C for 10 min.

## 1. Introduction

SmFe_12_-based alloys with the ThMn_12_-type crystal structure are expected to form a new generation of rare-earth permanent magnets due to their excellent intrinsic magnetic properties [1,2,3,4]. Compared with the high rare-earth content (11.8 at.%) of Nd_2_Fe_14_B permanent magnets, the rare-earth content in SmFe_12_-based permanent magnets is only 7.7 at.% [5,6]. However, the SmFe_12_ binary compound is thermodynamically unstable. Numerous experimental results and first-principles computational results have demonstrated that elements such as Ti, V, Al, Cr, Mo, Si and Mn can contribute to stabilizing the 1:12 main phase [7,8,9,10,11,12,13,14,15]. Among these stabilizing alloy elements, the Ti element is generally considered to have the best stabilizing effect and can effectively inhibit the precipitation of the α-Fe phase. At the same time, due to the high matching degree of the atomic size and lattice, the substitution content required to stabilize the 1:12 main phase must be as low as possible [16]. Srinithi et al. [17] reported that the addition of the V element can improve the coercivity (about 1.4 T) of a Sm_19.5_Fe_55_Al_12.5_Ti_5_V_8_ magnet because of the formation of the Sm-rich phase as a non-magnetic grain boundary phase [18]. The addition of V also reduces the lattice parameter c and the c/a ratio of the 1:12 main phase in the magnet to help stabilize the 1:12 main phase [19]. However, the addition of these stabilizing alloying elements will inevitably lead to a reduction in the magnetic properties of SmFe_12_-based permanent magnets. For example, Hirayama et al. [20] discovered that, as the Ti content increases, the intrinsic magnetic properties of Sm(Fe,Co)_12−*x*_Ti*_x_* compounds show marked deterioration. Some studies have shown that replacing part of the Fe with Co can improve this condition [12,20].

It has been reported that an important disadvantage of SmFe_12_-based permanent magnets is their lower coercivity. As an important magnetic performance parameter, the coercivity of the magnet determines the ability to maintain stability and resist external interference. The fine grain size method and the formation of the grain boundary phase are the main strategies to improve the coercivity (H_cj_) of SmFe_12_-based permanent magnets. Saito et al. [21] fabricated a SmFe_10_V_1.5_Ti_0.5_ magnet by using spark plasma sintering technology, with an average grain size of about 50 nm and coercivity of 5.2 kOe. Otsuka et al. [22] added excessive Sm elements to Sm(Fe,V)_12_ magnets, and the Sm-rich non-magnetic grain boundary phase (an amorphous structure with a composition ratio of Sm:(Fe + V) = 1:1) was formed in the magnets, uniformly surrounding the grains of the 1:12 main phase, and thus the coercivity of the magnets was significantly improved.

In recent years, some achievements have been made in replacing the Sm element to improve the magnetic properties of SmFe_12_-based alloys [14,16,23,24,25,26,27,28]. Replacing Sm with Zr helps to stabilize the 1:12 main phase in SmFe_12_-based alloys, which is associated with a reduction in the lattice constants [29]. The addition of light rare-earth elements Y and Ce can help to stabilize the 1:12 main phase and slightly improve the magnetic properties of the magnets [24,27]. Zhao et al. [30] reported that the core–shell structure of a Sm_0.75_Y_0.25_ (Fe_0.8_Co_0.2_)_11.25_Ti_0.75_ alloy was formed after a spontaneous harmonic decomposition process, which improved the inherent magnetic properties and stability of the alloy. Among rare-earth elements, La is a unique one. It cannot form binary compounds with Fe. It was confirmed by Gabay et al. [25] that the substitution of Zr by La in Zr_1−*x*_R*_x_*Fe_10_Si_2_ (R = Y, La, Ce, Pr and Sm) alloys avoided the appearance of the main phase with the ThMn_12_-type structure. The effects of La on the phase composition and magnetic properties of SmFe_11_Ti melt-spun ribbons were studied in our previous work [31]. The results indicated that the substitution of La for Sm was not conducive to the formation of the 1:12 main phase in SmFe_11_Ti ribbons. However, when a small amount of La was added to SmFe_11_Ti melt-spun ribbons, the magnetic properties of the ribbons were slightly improved. Therefore, the effect of La on alloys is of interest, since the co-existence of the 1:12 main phase and other phases is expected.

In addition, the magnetic properties of SmFe_12_-based permanent magnets can be improved by using heat treatments. Dirba et al. [12] showed that when the heat treatment temperature is low, the formation of the 1:12 main phase will be incomplete, while a high heat treatment temperature will lead to the decomposition of the 1:12 main phase and coarse grains. Zhou et al. [32] increased the coercive force of the Sm_1.7_Fe_10_V_2_ magnet to 10.6 kOe by adjusting the heat treatment time. It is suggested that the density of the magnet is low when the heat treatment time is low, but increasing the heat treatment time will lead to the significant evaporation of Sm.

In this work, Sm_1−*x*_La*_x_*Fe_10_V_2_ (*x* = 0, 0.2, 0.4) and SmLa*_y_*Fe_10_V_2_ (*y* = 0.1, 0.2, 0.3) ribbons were prepared using melt-spinning technology, and the effects of the La element on the phase composition and magnetic properties of these melt-spun ribbons were investigated by XRD and VSM. Furthermore, the magnetic properties of the SmLa_0.2_Fe_10_V_2_ ribbons were studied after different heat treatments.

## 2. Materials and Methods

Sm_1−*x*_La*_x_*Fe_10_V_2_ (*x* = 0, 0.2, 0.4) and SmLa*_y_*Fe_10_V_2_ (*y* = 0.1, 0.2, 0.3) alloys were synthesized via arc melting under a high-purity argon atmosphere via arc melting technology using pure metals Sm (pieces 99.9%), La (pieces 99.9%), Fe (particles 99.95%) and V (particles 99.9%). To compensate for Sm evaporation during arc melting, excessive amounts of Sm (5–10 wt.%) were added during metal smelting. The ingots were subsequently subjected to multiple remelting cycles (≥4 times) under argon to homogenize the composition and mitigate elemental segregation. By strictly controlling the process parameters to reduce the volatilization and oxidation of rare-earth elements during synthesis, the mass loss of the final alloy was less than 0.5 wt.%. After removing the oxides from the alloy surface, the alloy was loaded into a quartz tube with a pore size of 0.8 mm and melted under an argon atmosphere, and then the molten alloy was sprayed onto a rotating copper wheel at a wheel speed of 30 m/s to produce melt-spun ribbons. The melt-spun ribbons were encapsulated in a thin tantalum (Ta) foil and vacuum-sealed within a quartz glass tube. Subsequent annealing at 800 °C for 20 min was followed by rapid quenching in ice water.

The ribbons were annealed at 800 °C for 20 min and then ground in an agate mortar to obtain powders with particle sizes smaller than 200 mesh for XRD measurements. The phase structures of the ribbons were characterized by X-ray powder diffraction (XRD, PLXcel 3D, Tokyo, Japan, Cu K_α_ radiation, λ = 1.5406 Å, 45 kV, 40 mA, step = 0.013°) in the 2θ range of 20–70°. The magnetic properties of the ribbons were measured at room temperature using a vibrating sample magnetometer (VSM, Lakeshore Model 7400, Carson, CA, USA). The magnetic field range was between −20 kOe and 20 kOe. Since the external magnetic field was applied parallel to the ribbon plane, no demagnetization correction was applied to the ribbons.

## 3. Results and Discussion

### 3.1. Sm_1−x_La_x_Fe_10_V_2_ Ribbons

**Figure 1:**
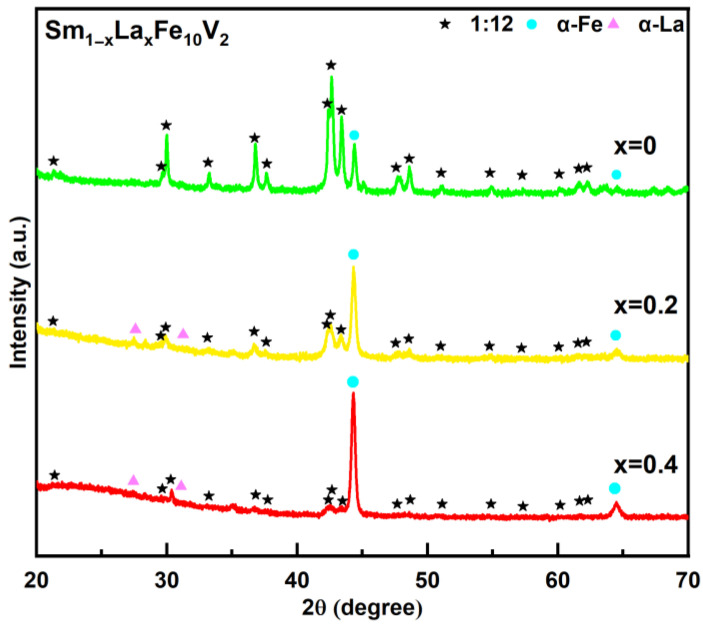
XRD patterns of Sm_1−*x*_La*_x_*Fe_10_V_2_ (*x* = 0, 0.2, 0.4) melt-spun ribbons after annealing at 800 °C for 20 min.

Figure 1 presents the phase formation revealed by the X-ray diffraction patterns in the Sm_1−*x*_La*_x_*Fe_10_V_2_ (*x* = 0, 0.2, 0.4) melt-spun ribbons after annealing at 800 °C for 20 min. The XRD patterns show that the ribbons consist of the 1:12 main phase with a ThMn_12_-type structure (space group I4/mmm), the soft-magnetic α-Fe phase (space group $Im\bar{3}m$) and the α-La phase (space group P6_3_/mmc). The XRD results show that the diffraction peaks of the Sm_1_Fe_10_V_2_ ribbon are less clear, and the spectral line is smooth, indicating the poor crystallinity of the 1:12 main phase, while the diffraction peak intensity of the α-Fe phase is low. The diffraction peak intensity of the 1:12 main phase in the Sm_0.8_La_0.2_Fe_10_V_2_ ribbon decreases rapidly, while the diffraction peak of the α-Fe phase increases and the α-La phase begins to appear. With a further increase in the substitution amount of La, the diffraction peaks of the α-Fe phase are basically visible in the XRD patterns of the Sm_0.6_La_0.4_Fe_10_V_2_ ribbon. These results indicate that the substitution of La for Sm is not conducive to the formation of the 1:12 main phase in Sm_1−*x*_La*_x_*Fe_10_V_2_ melt-spun ribbons. In studies of rare-earth element doping, Y and Ce are commonly used to help stabilize the 1:12 main phase. For example, Hagiwara et al. [33] achieved an almost single 1:12 main phase in a Sm(Fe,Co)_12−_*_x_* Ti*_x_* alloy by substituting Sm with Y. Liu et al. [28] demonstrated, through first-principles calculations, that the synergistic substitution of Ce and Zr could enhance the stability of the 1:12 main phase. Unlike these two rare-earth elements, in the study of the Zr_0.7_La_0.3_Fe_10_Si_2_ alloy by Gabay et al. [25], La could not replace Zr in the ZrFe_10_Si_2_ alloy. This may explain why La substitution for Sm fails to stabilize the 1:12 main phase.

**Figure 2:**
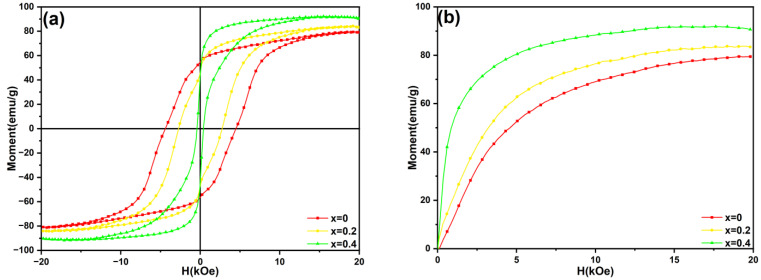
(**a**) Magnetic hysteresis loops and (**b**) initial magnetization curves of Sm_1−*x*_La*_x_*Fe_10_V_2_ (*x* = 0, 0.2, 0.4) melt-spun ribbons after annealing at 800 °C for 20 min.

**Figure 3:**
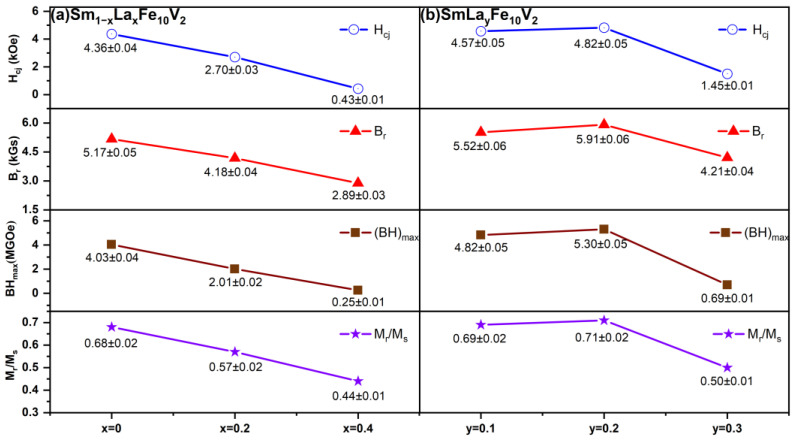
Magnetic properties of (**a**) Sm_1−*x*_La*_x_*Fe_10_V_2_ (*x* = 0, 0.2, 0.4) and (**b**) SmLa*_y_*Fe_10_V_2_ (*y* = 0.1, 0.2, 0.3) melt-spun ribbons after annealing at 800 °C for 20 min.

Figure 2 shows the hysteresis loops and initial magnetization curves of the Sm_1−*x*_La*_x_*Fe_10_V_2_ (*x* = 0, 0.2, 0.4) melt-spun ribbons after thermal annealing at 800 °C for 20 min. The maximum magnetic energy product ((BH)_max_) is determined by the maximum area of the rectangle in the second quadrant of the B-H curve converted from the measured M-H hysteresis loop. The corresponding magnetic properties of the ribbons are given in Table 1. In Figure 2a, it is shown that there is a slight kink in the Sm_1_Fe_10_V_2_ ribbon, which can be attributed to the exchange coupling between the hard magnetic 1:12 main phase and the soft magnetic α-Fe phase in the ribbon [34]. It can be seen from Figure 2b that the initial magnetization curve of the Sm_1_Fe_10_V_2_ ribbon increases rapidly under a low magnetic field. With an increase in the magnetic field, the magnetization tends to be saturated, indicating that the magnetization mechanism is regulated by the nucleation field [35]. However, with an increase in La substitution, the kink of the hysteresis loop in the ribbons becomes larger. This is because the substitution of La increases the phase content of the α-Fe phase in the ribbons. With an increase in the phase content of the α-Fe phase, the exchange coupling between the hard magnetic phase and the soft magnetic phase in the ribbons would be disrupted. This is consistent with the XRD results shown in Figure 1. Figure 3 illustrates the variations in the coercivity (H_cj_), remanence (B_r_), maximum magnetic energy product ((BH)_max_) and remanence ratio (M_r_/M_s_) of Sm_1−*x*_La*_x_*Fe_10_V_2_ (*x* = 0, 0.2, 0.4) melt-spun ribbons with the substitution of La for Sm. The remanence ratio (M_r_/M_s_) can explain the exchange coupling in the ribbons. In general, the ribbon exhibits a remanence enhancement effect when the remanence ratio exceeds 0.5. It can be seen from Figure 3 that the remanence ratios of the ribbons decrease significantly with the increasing substitution of Sm by La, indicating that the substitution of La would destroy the exchange coupling in the ribbons.

The experimental results mentioned above demonstrate that substituting La for Sm significantly inhibits the formation of the 1:12 main phase in the ribbons, while promoting a substantial increase in the phase content of the α-Fe phase. Magnetic property measurements indicate that the magnetization mechanism is governed by the nucleation field. With increasing La substitution, the coercivity of the ribbons decreases from 4.36 kOe to 0.43 kOe, and the maximum magnetic energy product declines from 5.17 MGOe to 2.89 MGOe. This deterioration is attributed to a significant reduction in the phase content of the 1:12 main phase in the ribbons.

### 3.2. SmLa_y_Fe_10_V_2_ Ribbons

**Figure 4:**
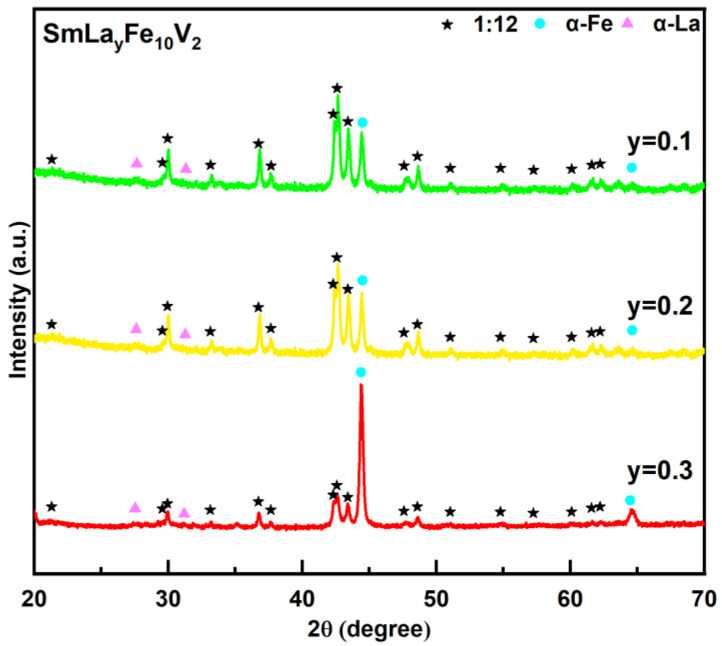
XRD patterns of SmLa*_y_*Fe_10_V_2_ (*y* = 0.1, 0.2, 0.3) melt-spun ribbons after annealing at 800 °C for 20 min.

It was observed that the substitution of Sm by La plays a serious negative role in the formation of the 1:12 main phase, resulting in a decrease in the magnetic properties of the Sm_1−*x*_La*_x_*Fe_10_V_2_ (*x* = 0, 0.2, 0.4) melt-spun ribbons. Thus, ribbons with additional La elements were investigated experimentally in this work. Figure 4 shows the XRD patterns of the SmLa_y_Fe_10_V_2_ (*y* = 0.1, 0.2, 0.3) melt-spun ribbons. As can be seen in Figure 4, the ribbons are still composed of the 1:12 main phase (space group I4/mmm), the soft magnetic α-Fe phase (space group $Im\bar{3}m$) and the α-La phase (space group P6_3_/mmc). When a small amount of La is added, the formation of the 1:12 main phase is not significantly affected in the ribbons. Meanwhile, the diffraction peak shape of the 1:12 main phase is clearer, which indicates that the crystallinity of the 1:12 main phase in the ribbons is improved. The relative intensity of the diffraction peaks of the α-Fe phase in the ribbons also increases slightly. The XRD patterns of the SmLa*_y_*Fe_10_V_2_ ribbon with *y* = 0.3 show that the relative intensity of the diffraction peaks of the α-Fe phase increases significantly compared with the 1:12 main phase. This indicates a significant increase in phase content for the α-Fe phase. The results show that the phase content of the α-Fe phase increases greatly when the added amount of La increases further in the ribbons.

**Figure 5:**
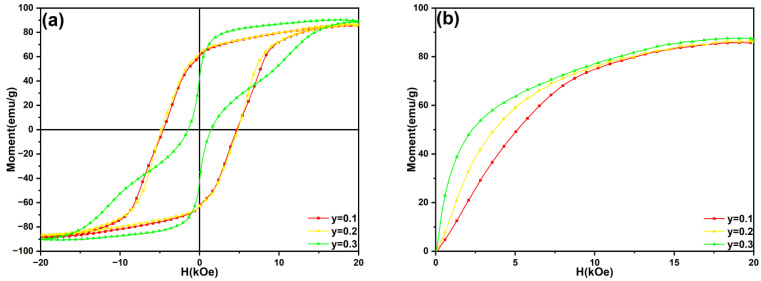
(**a**) Magnetic hysteresis loops and (**b**) initial magnetization curves of SmLa_*y*_Fe_10_V_2_ (*y* = 0.1, 0.2, 0.3) melt-spun ribbons after annealing at 800 °C for 20 min.

Figure 5 shows the hysteresis loops and initial magnetization curves of the SmLa*_y_*Fe_10_V_2_ (*y* = 0.1, 0.2, 0.3) melt-spun ribbons. The variation trends of the coercivity (H_cj_), remanence (B_r_), maximum magnetic energy product ((BH)_max_) and remanence ratio (M_r_/M_s_) of the ribbons are shown in Figure 3. It reveals that a small amount of La (*y* ≤ 0.2) addition increases the remanence ratio (M_r_/M_s_) of the ribbons. The reason for the increase in the magnetic properties may be that the addition of La reduces the grain size in the ribbons and improves the exchange coupling between grains; with the increase in La addition, the coercivity increases firstly and then decreases. A similar trend in the remanence and maximum magnetic energy products of the ribbons was also observed. When *y* = 0.3 in SmLa*_y_*Fe_10_V_2_ ribbons, a large amount of the α-Fe phase is precipitated, which seriously affects the exchange coupling and leads to low coercivity. Finally, the coercivity of the SmLa_0,2_Fe_10_V_2_ ribbon reaches the maximum (H_cj_ = 4.82 kOe).

In our previous work [31], the influence of La on the phase composition and magnetic properties of SmFe_11_Ti ribbons was investigated. The results obtained were similar to those in this study. However, the difference is that, when La is slightly substituted, the 1:12 main phase is still dominant in the ribbons, and the phase fraction of the α-Fe phase is relatively small. As shown in research on Sm_1−*x*_La*_x_*Fe_10_V_2_ ribbons, even a small amount of La substitution (*x* = 0.2) can lead to a significant reduction in the 1:12 main phase in the ribbons and the large precipitation of the α-Fe phase. The magnetic properties of both ribbons also exhibited similar trends, with the coercivity reaching optimal values when the La substitution reached 0.2. Specifically, the coercivity of the SmLa*_y_*Fe_11_Ti ribbons increased from 4.21 kOe to 4.56 kO. The TEM analysis of the SmLa_0.2_Fe_11_Ti ribbon revealed that the α-La phase did not exist as an intergranular phase at the grain boundaries but instead coexisted with the 1:12 main phase as a separate secondary phase. Based on this observation, it is concluded that La incorporated into a SmFe_10_V_2_ ribbon is unlikely to form an intergranular phase.

The experimental results in this work indicate that minor La addition (*y* ≤ 0.2) does not suppress the formation of the 1:12 main phase in the ribbons. However, when the La content reaches y = 0.3, the 1:12 main phase is significantly inhibited, accompanied by the substantial precipitation of the α-Fe phase. Magnetic property measurements reveal that, with La addition, the coercivity of the ribbons initially increases from 4.32 kOe to 4.82 kOe and subsequently decreases to 1.45 kOe, while the maximum magnetic energy product rises from 4.03 MGOe to 5.30 MGOe before dropping sharply to 0.69 MGOe. The initial enhancement in magnetic performance can be attributed to the minor La addition, which refines the grain size of the ribbons and improves the intergranular exchange coupling. However, a further increase in La addition suppresses the formation of the hard magnetic 1:12 main phase while inducing the excessive precipitation of the soft magnetic α-Fe phase, ultimately leading to the deterioration of the magnetic performance.

### 3.3. SmLa_0.2_Fe_10_V_2_ Ribbons

According to the obtained magnetic properties of the SmLa*_y_*Fe_10_V_2_ (*y* = 0.1, 0.2, 0.3) melt-spun ribbons, it was noted that the SmLa_0.2_Fe_10_V_2_ ribbon exhibited better magnetic properties, and thus the phase formation and magnetic properties of ribbons under different heat treatment conditions were studied further experimentally in this work.

**Figure 6:**
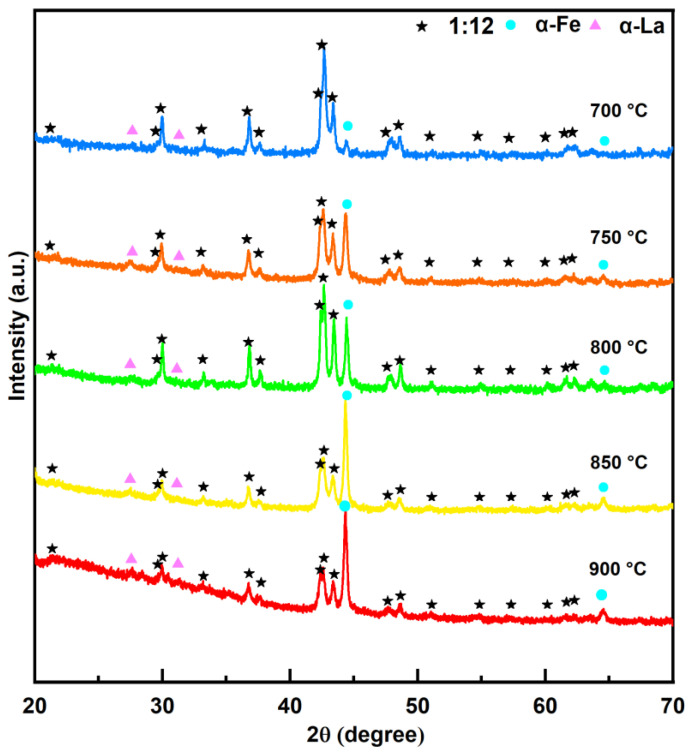
XRD patterns of SmLa_0.2_Fe_10_V_2_ melt-spun ribbons after annealing at different temperatures for 20 min.

Figure 6 shows the XRD patterns of the SmLa_0.2_Fe_10_V_2_ ribbons after annealing at different temperatures for 20 min. The XRD results show that the ribbon consists of the 1:12 main phase (space group I4/mmm) with the ThMn_12_ structure, the soft magnetic α-Fe phase (space group $Im\bar{3}m$) and the α-La phase (space group P6_3_/mmc). When the ribbon is annealed at 700 °C, the intensity of the diffraction peaks of the 1:12 main phase is weak, indicating that the crystallinity of the 1:12 main phase is not perfect. The intensity of the diffraction peaks of the α-Fe phase is much lower compared with those of the 1:12 main phase, indicating that the phase content of the α-Fe phase in the ribbon is small. With an increase in the annealing temperature, the intensity of the diffraction peaks of the 1:12 main phase becomes gradually sharper, indicating that the crystallinity of the main phase in the ribbon is gradually improved. Meanwhile, it was observed that, compared to the 1:12 main phase, the relative diffraction intensity of the α-Fe phase increases significantly with the elevation of the annealing temperature. This indicates that the phase fraction of the α-Fe phase increases. According to the report of Chen et al. [36], this is because the crystallization of the amorphous phase increases the phase fraction of the α-Fe phase. These results mean that an increase in the annealing temperature is beneficial to the crystallization of the 1:12 main phase, while a higher annealing temperature leads to the rapid formation of the α-Fe phase in the ribbons.

**Figure 7:**
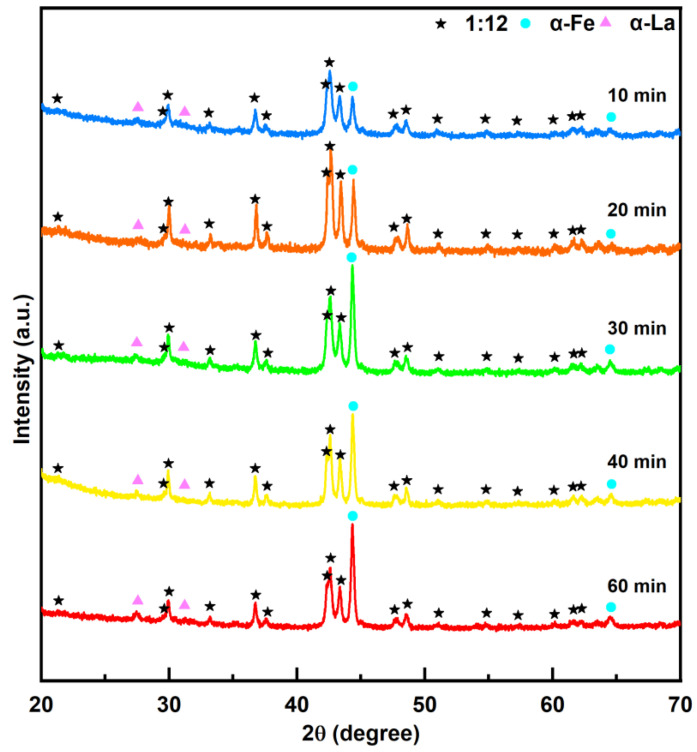
XRD patterns of SmLa_0.2_Fe_10_V_2_ melt-spun ribbons after annealing at 800 °C for different times.

Figure 7 shows the XRD patterns of the SmLa_0.2_Fe_10_V_2_ melt-spun ribbons after annealing at 800 °C for different times. Upon increasing the annealing time at 800 °C, the sharpness of the XRD diffraction peaks of the ribbon increased, indicating an improvement in the crystallinity of the 1:12 main phase. When the annealing time is short, the relative intensities of the diffraction peaks of the 1:12 main phase and the α-Fe phase show no significant changes. When the annealing time exceeded 30 min, the relative intensity of the diffraction peaks of the α-Fe phase increased greatly. These results show that the phase content of the α-Fe phase increases in the ribbon with an increase in the annealing time.

**Figure 8:**
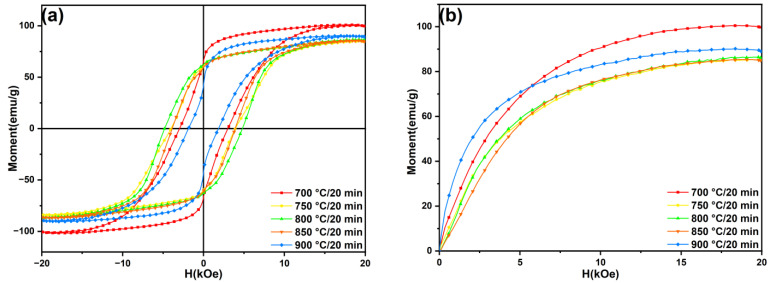
(**a**) Magnetic hysteresis loops and (**b**) initial magnetization curves of SmLa_0.2_Fe_10_V_2_ melt-spun ribbons after annealing at different temperatures for 20 min.

**Figure 9:**
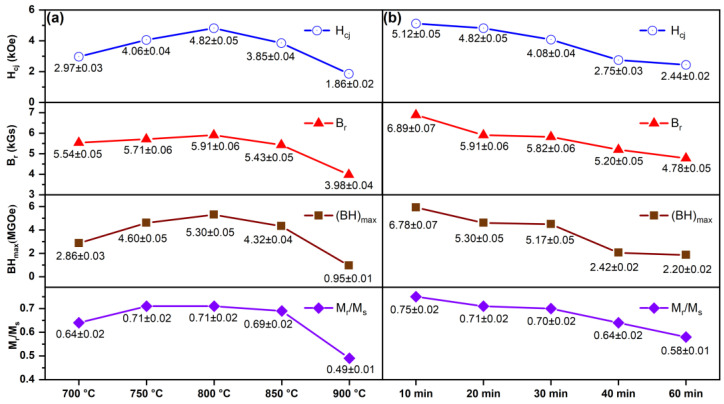
(**a**) Magnetic properties of SmLa_0.2_Fe_10_V_2_ melt-spun ribbons after annealing (**a**) at different temperatures for 20 min and (**b**) at 800 °C for different times.

Figure 8 shows the hysteresis loops and initial magnetization curves of the SmLa_0.2_Fe_10_V_2_ melt-spun ribbons after annealing at different temperatures for 20 min. The measured magnetic properties (H_cj_, B_r_, (BH)_max_ and M_r_/M_s_) of the ribbons after different heat treatments in this work are shown in Table 2. Figure 9 displays the variation trends of the magnetic properties of the ribbons. In Figure 8a, the demagnetization curve of the ribbon after annealing at 700 °C presents a relatively smooth profile in the second quadrant, indicating typical single-phase hard magnetic behavior. It can be seen that the ribbon contains mainly the 1:12 main phase after annealing at a low temperature, and the phase content of the α-Fe phase is lower. With an increase in the annealing temperature, the phase content of the α-Fe phase in the ribbon increases. The phase content of the α-Fe phase in the ribbon becomes too high, and thus the exchange coupling effect in the ribbon is affected. It can be seen from Figure 9 that the magnetic properties of the ribbon show a trend of first increasing and then decreasing with the increase in the annealing temperature. Lee et al. [37] reported that the coercivity of Sm*ₓ*Fe_11_Ti (*x* = 0.9, 1.0 and 1.1) ribbons subjected to heat treatments at different temperatures (600–950 °C) exhibited a trend of initially increasing and then decreasing, with the maximum coercivity of 6.3 kOe achieved at an annealing temperature of 850 °C. Their TEM analysis of SmFe_11_Ti ribbons revealed that the grain size in the ribbons was approximately 40 nm at 850 °C but increased to 260 nm when the heat treatment temperature was raised to 950 °C. Clearly, excessive heat treatment temperatures lead to the significant coarsening of the grain size. In the present study, the optimal magnetic properties were achieved in the ribbon annealed at 800 °C for 20 min. This phenomenon can be attributed to the enhanced crystallinity of the 1:12 main phase in the ribbons, combined with an appropriate grain size and effective intergranular exchange coupling.

**Figure 10:**
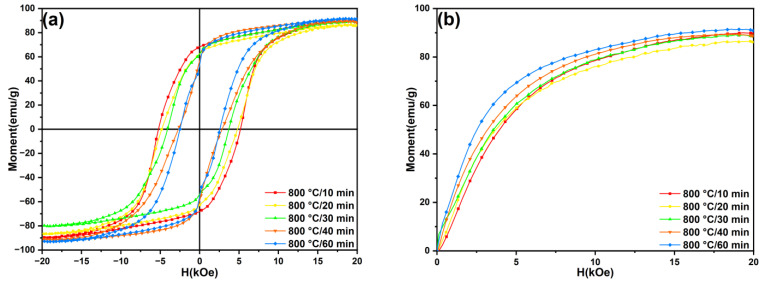
(**a**) Magnetic hysteresis loops and (**b**) initial magnetization curves of SmLa_0.2_Fe_10_V_2_ melt-spun ribbons after annealing at 800 °C for different times.

Figure 10 shows the hysteresis loops and initial magnetization curves of the SmLa_0.2_Fe_10_V_2_ melt-spun ribbons after annealing at 800 °C for different times. The variation trends in the magnetic properties and specific data are given in Figure 9 and Table 2. From Figure 10a, we can observe that the saturation magnetization (M_s_) of the ribbons remains relatively consistent under different heat treatment times, but the coercivity (H_cj_) of the ribbons shows an obvious dependence on the heat treatment time. From Figure 9, it can be found that the coercivity of the ribbon at 800 °C shows a monotonically decreasing trend with the increase in the annealing time. This is because, with an increase in the annealing time, the phase content of the α-Fe phase increases, while that of the 1:12 main phase decreases. It was reported by Zhou et al. [32] that when the heat treatment time exceeded 40 min, prolonged exposure to elevated temperatures caused the significant volatilization of Sm, leading to the partial decomposition of the 1:12 main phase, which subsequently resulted in the degradation of the magnetic properties of the alloy. Finally, the magnetic properties of the ribbons reached their optimum values (H_cj_ = 5.12 kOe, B_r_ = 6.89 kGs, M_r_/M_s_ = 0.75 and (BH)_max_ = 6.78 MGOe) after annealing at 800 °C for 10 min.

The experimental results in this work indicate that all ribbons consist of the 1:12 main phase, α-Fe phase and α-La phase. The magnetic measurement results reveal that, as the annealing temperature increases, the coercivity initially increases from 2.97 kOe to 4.82 kOe and then decreases to 1.86 kOe, while the maximum magnetic energy product first rises from 5.54 MGOe to 5.91 MGOe before declining to 3.98 MGOe. This trend arises because the crystallinity of the 1:12 main phase in the ribbons gradually improves with temperature elevation, thereby enhancing the magnetic properties. However, when the temperature further increases, the magnetic performance deteriorates due to grain coarsening (which weakens intergranular exchange coupling) and the excessive precipitation of the soft magnetic α-Fe phase within the ribbons. With prolonged heat treatment, the coercivity and maximum magnetic energy products of the ribbons exhibit a decreasing trend. This is attributed to increased Sm volatilization, which induces the partial decomposition of the main phase, coupled with the enhanced content of the α-Fe phase in the ribbons.

## 4. Conclusions

The effects of the La element and heat treatment on the phase formation and magnetic properties of Sm_1−*x*_La*_x_*Fe_10_V_2_ and SmLa*_y_*Fe_10_V_2_ melt-spun ribbons were studied experimentally. The following conclusions could be drawn.

(1) The XRD results demonstrate that both Sm_1−*x*_La*_x_*Fe_10_V_2_ (*x* = 0, 0.2, 0.4) and SmLa*_y_*Fe_10_V_2_ (*y* = 0.1, 0.2, 0.3) ribbons are composed of the 1:12 main phase with a ThMn_12_-type structure, α-Fe phase and α-La phase. Substituting La for Sm induces a gradual reduction in the phase fraction of the 1:12 main phase and an increase in the α-Fe phase content, thereby leading to the deterioration of the magnetic properties. Minor La addition (y ≤ 0.2) exhibits no significant impact on the formation of the 1:12 main phase in SmLa*_y_*Fe_10_V_2_ ribbons.
(2) Heat treatment experiments on SmLa_0.2_Fe_10_V_2_ ribbons reveal that higher annealing temperatures improve the crystallinity of the 1:12 main phase. Excessively high annealing temperatures or prolonged annealing durations promote the precipitation of the α-Fe phase, resulting in the deterioration of the magnetic properties. After annealing at 800 °C for 10 min, the optimal magnetic properties (H_cj_ = 5.12 kOe, B_r_ = 6.89 kGs, (BH)_max_ = 6.78 MGOe) of the SmLa_0.2_Fe_10_V_2_ ribbon were obtained.

## Figures and Tables

**Table 1:** Magnetic properties of Sm_1−*x*_La*_x_*Fe_10_V_2_ and SmLa*_y_*Fe_10_V_2_ melt-spun ribbons after annealing at 800 °C for 20 min.

Ribbon	H_cj_ (kOe)	B_r_ (kGs)	(BH)_max_ (MGOe)	M_r_/M_s_
SmFe_10_V_2_	4.36 ± 0.04	5.17 ± 0.05	4.03 ± 0.04	0.68 ± 0.02
Sm_0.8_La_0.2_Fe_10_V_2_	2.70 ± 0.03	4.18 ± 0.04	2.01 ± 0.02	0.57 ± 0.02
Sm_0.6_La_0.4_Fe_10_V_2_	0.43 ± 0.01	2.89 ± 0.03	0.25 ± 0.01	0.44 ± 0.01
SmLa_0.1_Fe_10_V_2_	4.57 ± 0.05	5.52 ± 0.06	4.82 ± 0.05	0.69 ± 0.02
SmLa_0.2_Fe_10_V_2_	4.82 ± 0.05	5.91 ± 0.06	5.30 ± 0.05	0.71 ± 0.02
SmLa_0.3_Fe_10_V_2_	1.45 ± 0.01	4.21 ± 0.04	0.69 ± 0.01	0.50 ± 0.01

**Table 2:** Magnetic properties of SmLa_0.2_Fe_10_V_2_ melt-spun ribbons after different heat treatments.

Heat Treatment	H_cj_ (kOe)	B_r_ (kGs)	(BH)_max_ (MGOe)	M_r_/M_s_
700 °C/20 min	2.97 ± 0.03	5.54 ± 0.05	2.86 ± 0.03	0.64 ± 0.02
750 °C/20 min	4.06 ± 0.04	5.71 ± 0.06	4.60 ± 0.05	0.71 ± 0.02
800 °C/20 min	4.82 ± 0.05	5.91 ± 0.06	5.30 ± 0.05	0.71 ± 0.02
850 °C/20 min	3.85 ± 0.04	5.43 ± 0.05	4.32 ± 0.04	0.69 ± 0.02
900 °C/20 min	1.86 ± 0.02	3.98 ± 0.04	0.95 ± 0.01	0.49 ± 0.01
800 °C/10 min	5.12 ± 0.05	6.89 ± 0.07	6.78 ± 0.07	0.75 ± 0.02
800 °C/30 min	4.08 ± 0.04	5.82 ± 0.06	5.17 ± 0.05	0.70 ± 0.02
800 °C/40 min	2.75 ± 0.03	5.20 ± 0.05	2.42 ± 0.02	0.64 ± 0.02
800 °C/60 min	2.44 ± 0.02	4.78 ± 0.05	2.20 ± 0.02	0.58 ± 0.01

## Data Availability

The original contributions presented in the study are included in the article, further inquiries can be directed to the corresponding author.
